# Advancing Immediate Breast Reconstruction Surgery in Pakistan: Bridging Literature Gaps and Meeting Patient Needs

**DOI:** 10.1055/a-2312-8945

**Published:** 2024-10-22

**Authors:** Abdullah Bin Faisal, Fatima Shahid, Laiba Khalid, Mohammad Fazlur Rahman

**Affiliations:** 1 Medical College, The Aga Khan University, Karachi, Sindh, Pakistan; 2 Medical College, King Edward Medical College, Lahore, Punjab, Pakistan; 3 Department of Surgery, The Aga Khan University, Karachi, Sindh, Pakistan

Breast cancer is now the most frequently diagnosed cancer, with 2.26 million new cases in 2020, and it ranks as the leading cause of cancer mortality in women globally. 1 In Pakistan, it stands as the predominant cancer among females, affecting nearly one in nine women. Its incidence in Pakistan surpasses that of neighboring countries like Iran and India by 2.5 times. 2 Stigmatization and societal pressures, coupled with a reluctance to address breast cancer, hinder early detection and treatment in low- and middle-income countries. Physical barriers further exacerbate psychosocial stress, deterring patients from pursuing screening and treatment. In Pakistan, limited research has explored factors contributing to delays, with awareness gaps being a notable concern. 3 Unfortunately, Pakistan lags significantly behind in embracing this vital aspect of breast cancer care. Among the treatment options available to patients, mastectomy is a critical and often life-saving intervention. However, the postmastectomy phase presents a unique challenge: how to restore both physical and psychosocial well-being. Breast reconstruction surgery, specifically immediate breast reconstruction (IBR), has gained prominence in many developed countries as a means to address this challenge. Shaker et al demonstrated close to 90% day-case success rate for mastectomy with IBR. 4

A 2016 study conducted at The Aga Khan University offered a glimpse into the state of breast reconstruction surgery in Pakistan. “Breast reconstruction at The Aga Khan University—A 10-year audit” by Abdullah et al (2016) sheds light on the scarcity of this procedure in Pakistan. While reconstruction rates vary globally from 5 to 50%, anecdotal evidence suggests that less than 1% of Pakistani women opt for reconstruction. 5 Literature gaps in this area remain substantial, leaving Pakistani breast cancer patients with limited choices and inadequate access to comprehensive care.

However, there is hope on the horizon. “Patient-reported outcomes for IBR with mastectomy among breast cancer patients in Pakistan,” a 2022 prospective comparative study by Afzal et al, provides a glimmer of insight into the patient perspective regarding IBR in Pakistan. 6 The study highlights the positive impact of IBR on patient satisfaction with their breasts and psychosocial well-being. It signifies a step in the right direction, demonstrating that better health-related outcomes are reported by patients undergoing IBR. Furthermore, this research shows that IBR should be offered routinely to patients undergoing mastectomy in Pakistan, irrespective of their socioeconomic and educational status.

Nonetheless, several critical literature gaps persist in this field in Pakistan, hindering the development of effective breast reconstruction strategies and policies. To truly advance breast reconstruction surgery in the country, it is imperative that these gaps are addressed.

1. Comprehensive National Data: While the study by Abdullah et al provides valuable insights, a comprehensive, nationwide audit of breast reconstruction surgeries is urgently needed. This would allow us to understand the extent of the issue and help policymakers allocate resources appropriately. 5 Additionally, it would facilitate international comparisons to identify best practices that can be implemented in Pakistan.
2. Cultural and Awareness Barriers: Pakistan faces unique cultural and societal barriers that deter women from considering breast reconstruction. A deeper exploration of these barriers and targeted awareness campaigns are essential to increase patient acceptance of IBR. 3
3. Access and Education: Limited access to reconstructive services and a lack of awareness among health care providers are significant hurdles. Medical institutions and professional organizations should prioritize training programs and workshops to equip surgeons with the skills needed for IBR. 7
4. Patient-centered Care: While the study by Afzal et al provides valuable patient-reported outcomes, more research is needed to explore the long-term physical and psychosocial effects of IBR in the Pakistani context. 6 Understanding patient perspectives, including their concerns and expectations, is crucial for tailoring care to their needs.

In conclusion, while recent research offers glimpses into the world of breast reconstruction surgery in Pakistan, we must recognize the existing literature gaps and address them urgently. It is imperative to conduct more comprehensive studies, tackle cultural barriers, improve access and education, and prioritize patient-centered care. By doing so, we can ensure that all Pakistani breast cancer patients have access to the best possible care, ultimately improving their quality of life and survivorship. Let us embark on this journey to transform the landscape of breast reconstruction surgery in Pakistan and provide a brighter future for our patients.
